# Stereotactic body radiation therapy and radioembolization as bridging strategies to liver transplantation: a retrospective real-world analysis

**DOI:** 10.3389/ti.2026.17047

**Published:** 2026-10-14

**Authors:** Nicola Colucci, Mitchell A. Webb, Muhammad Uzair Khalid, Anastasios D. Giannou, Yuwei Yang, Tyler D. Yan, Lok In Josephine Ma, Ravjot Dhatt, Stéphanie Chartier-Plante, Peter T. W. Kim, Graziano Oldani, Maja Segedi, Roy M. K. Ma, Stephen G. F. Ho, Emma M. Dunne, Michael S. Bleszynski

**Affiliations:** 1 Department of Surgery, University of British Columbia, Vancouver, BC, Canada; 2 Department of Radiology, University of British Columbia, Vancouver, BC, Canada; 3 Faculty of Medicine, University of British Columbia, Vancouver, BC, Canada; 4 Department of Radiation Oncology, British Columbia Cancer Agency, Vancouver, BC, Canada

**Keywords:** hepatocellular carcinoma, liver transplantation, radiation oncology, stereotactic radiation therapy, therapeutic radioembolization

Dear Editors,

Liver transplantation (LT) remains the most effective curative option for selected patients with hepatocellular carcinoma (HCC), addressing both tumor burden and the underlying liver disease. Selection has evolved beyond the original Milan criteria toward a more dynamic framework, incorporating tumor biology and response to therapy [1]. Within this paradigm, locoregional treatments (LRT) are not only used to maintain eligibility, but also to inform candidacy over time. Radiation-based therapies, including yttrium-90 transarterial radioembolization (TARE) and stereotactic body radiation therapy (SBRT), are increasingly integrated into these pathways. Both provide high rates of local control and are used as bridging or downstaging strategies [2, 3]; treatment choice is typically individualized, depending on tumor burden, vascular anatomy, hepatic functional reserve, prior treatments and center expertise.

We performed a retrospective analysis of all adult patients undergoing LT for HCC at Vancouver General Hospital between 2015 and 2023, who received either TARE or SBRT as their final radiation treatment before transplantation. Prior LRTs were allowed, reflecting current clinical practice. Liver and patient safety was assessed by monitoring the MELD score dynamics pre- and post-treatment, as well as the occurrence of Radiation-Induced Liver Disease (RILD). Radiographic response of treated lesions was classified according to the LI-RADS Radiation Treatment Response (LR-TR) algorithm [4], using imaging acquired within 6 months following radiation. All pre-transplant imaging were systematically reviewed by a dedicated radiologist and a transplant surgeon, with final consensus provided by a senior abdominal radiologist. Total tumor volume (TTV) was calculated using CT or MRI, assuming spherical geometry from lesions classified as LI-RADS 4 or 5. Pre-transplant imaging corresponded to the most recent study within 6 months prior to transplantation, and was used to obtain updated pre-transplant TTV and LR-TR of the treated lesions. Explant pathology was reviewed to determine pathologic complete response (pCR) of the treated lesions and calculate viable TTV.

A total of 56 patients met inclusion criteria: 23 received TARE and 33 received SBRT. Baseline demographics were similar between groups, including age (61.7 vs. 60.7 years, p = 0.57), sex distribution (87% male in both) and underlying liver disease. Treatment allocation was determined by a multidisciplinary Liver Tumor Board: TARE was preferentially selected for patients with a higher baseline tumor burden, larger maximum tumor diameter (3.8 vs. 3.2 cm, p = 0.014) and greater TTV (29.6 vs. 17.9 cm^3^, p < 0.01). Conversely, SBRT was more frequently used as a salvage modality following prior multimodal LRT (79% SBRT vs. 35% TARE, p < 0.01). It was also favored for lesions in anatomically challenging positions, or in patients for whom further surgical, ablative, or transarterial therapy was considered unsuitable. SBRT was delivered per institutional practice [5, 6]. The gross tumor volume (GTV) was defined as arterial-enhancing lesions with delayed washout on co-registered diagnostic imaging, with an isotropic 5 mm clinical target volume (CTV) margin confined to the liver parenchyma. Motion management included dynamic tumor tracking (DTT), respiratory gating, or internal target volume strategies based on individual patient suitability. Daily cone-beam CT guidance was performed for non-gated or non-DTT cases. Planning strictly prioritized organ-at-risk constraints over target volume coverage. The median prescribed dose was 45 Gy (range 35–45 Gy) in 3–5 fractions, corresponding to a median biologically effective dose (BED_10_) of 112.5 Gy_10_.

On post-treatment imaging, non-viable disease was reported in 52.2% of TARE and 57.6% of SBRT-treated lesions, while viable disease persisted in 30.4% and 15.2% (p = 0.35).

Both approaches were well tolerated. Median MELD prior to treatment was 8 in both groups, with minimal change following therapy (ΔMELD = 1.0 in both, p = 0.92). There was one suspected case of RILD following SBRT, presenting as hepatic decompensation. The median interval from final radiation treatment to liver transplantation was comparable between groups (337 days for SBRT vs. 360 days for TARE, p = 0.742). Post-transplant outcomes, including surgical complications and length of stay, were comparable between groups, indicating that pre-transplant radiation did not adversely affect surgical feasibility or recovery.

Explant analysis demonstrated variable residual tumor burden. pCR was observed in 36.4% of SBRT and 21.7% of TARE-treated lesion (p = 0.38). Although pCR was numerically higher after SBRT, this possibly reflects a cumulative treatment effect following prior therapies and the lower baseline tumor burden in this cohort, rather than implying the superiority of a specific modality. Overall radiographic-pathologic discordance was nearly identical between modalities (40.6% for SBRT vs. 40.9% for TARE, p > 0.99). Among lesions classified as non-viable on repeated pre-transplant imaging, only 58% demonstrated complete necrosis on explant, despite an overall predictive OR of 4.22 (1.06–19.52, p = 0.037). Neither subgroup individually demonstrated a statistically significant association between LR-TR classification and pathologic viability (SBRT: OR 2.93, 0.55–17.07, p = 0.28; TARE: OR 3.75, 0.23–132.89, p = 0.59), confirming that radiographic overestimation of complete necrosis occurs across both treatment pathways. Similarly, there was no meaningful correlation between radiographic and pathologic TTV throughout the cohort, which accounted for the total burden of treated and untreated lesions. Although a stronger Pearson’s correlation was observed in the SBRT group (r = 0.76, p < 0.001) compared to TARE (r = −0.07, p = 0.77) (Supplementary Figure S1), this is most likely a reflection of the lower overall tumor burden in the SBRT group. These findings underscore the limitations of cross-sectional imaging in the post-radiation setting, where treatment-induced fibrosis and inflammatory changes can mask viable tumor.

Alpha-fetoprotein (AFP) kinetics were also not predictive of pCR. In multivariable analysis adjusting for baseline AFP, tumor burden and Milan status, the AFP log-ratio was not associated with pCR (OR 1.13, 0.43–2.92, p = 0.81). This reinforces existing concerns that AFP may be an imperfect surrogate for complete tumor eradication in the post-radiation setting. Post-transplant outcomes were favorable over a comparable median follow-up (35 months for SBRT vs. 36 for TARE, p = 0.884). Recurrence-free survival at 1 and 3 years was 91.3% and 77.1% for TARE and 93.9% and 76.7% for SBRT, respectively. In an adjusted Cox model, treatment modality was not associated with post-transplant recurrence (HR 1.49, 0.38–5.80, p = 0.56). Overall survival at 1 and 3 years was similarly high, with no significant differences between groups (Figure 1). Further details about patient demographics, baseline and pre-transplant tumor characteristics, prior therapies, post-treatment responses, surgical data, and explant pathology findings are detailed in Supplementary Material 1.

**FIGURE 1:**
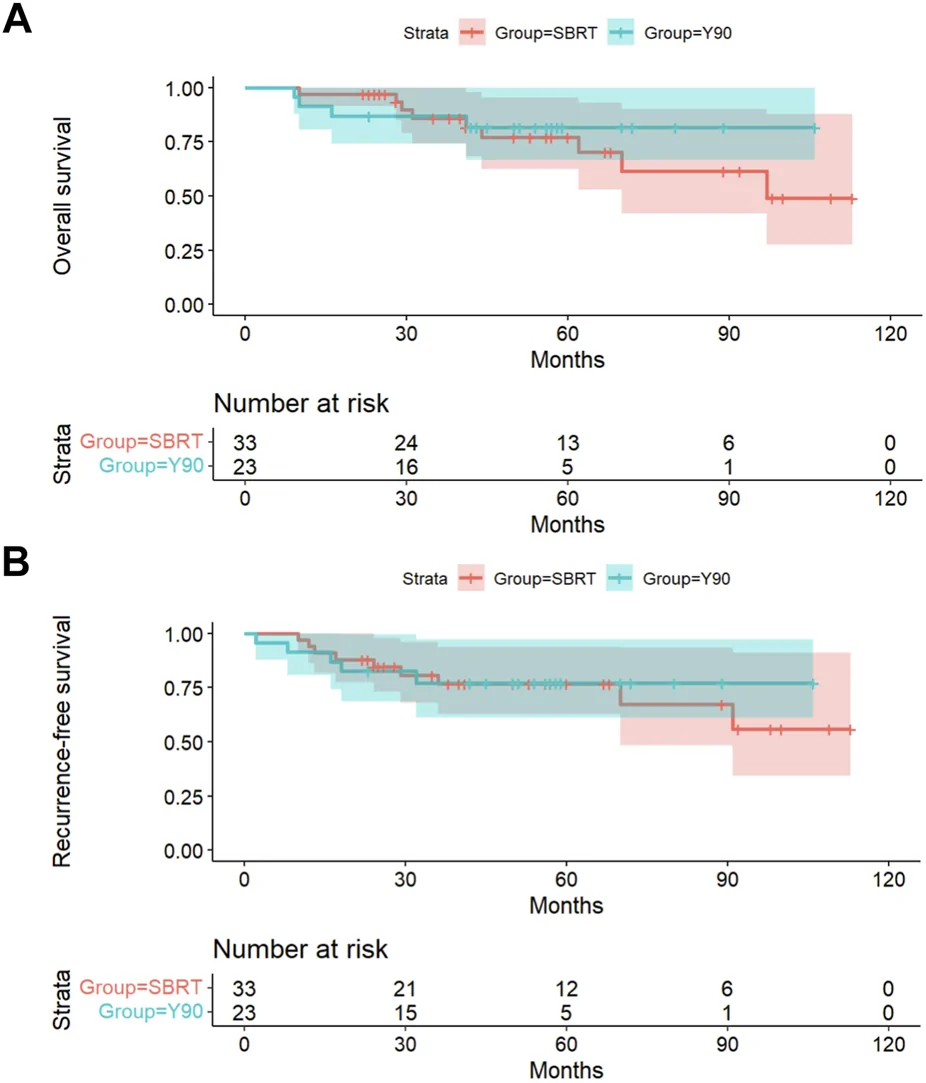
Kaplan-Meier estimates of **(A)** overall survival and **(B)** recurrence-free survival in patients undergoing liver transplantation following SBRT (red) or TARE (cyan).

This study reflects real-world use of radiation-based therapies in LT candidates with HCC. Rather than functioning as isolated interventions, TARE and SBRT are applied within iterative, multimodal treatment pathways. TARE was preferentially used in patients with higher initial tumor burden, while SBRT was commonly employed after multiple treatments. Within these distinct treatment pathways, both modalities were safe and associated with favorable post-transplant outcomes. Our observed per-lesion pathologic complete response rates reflect real-world bridging in a pre-treated cohort. For SBRT, our pCR rate is lower than the 58.3% per-lesion rate reported in prospective trial data [7], which exclusively evaluated treatment-naïve candidates. For TARE, while highly selective ablative segmentectomy series report pCR rates of 68% [8], our 21.7% rate aligns with real-world national registry data reporting an overall pCR rate of 33.6%, and a center-level range of 0.0%–50.0% [9]. The differences across studies likely reflect differences in patient selection, baseline tumor burden, prior LRT exposure, treatment technique, and definitions of pathologic response. Radiographic post-treatment assessment was frequently discordant with explant findings: non-viability often overestimated complete tumor necrosis, and neither AFP kinetics nor imaging reliably predicted pCR. This has direct implications for waitlist management and underscores the need for continued surveillance and cautious interpretation of treatment response. Emerging tools, including circulating tumor DNA and radiomics, might eventually provide more granular data to improve prediction of tumor burden and recurrence before and after transplantation [10]. The limitations of this study include its retrospective design, modest sample size, and the inherent treatment selection bias associated with multimodal therapy. Additionally, restricting volumetric analysis to LI-RADS 4/5 lesions and the interval between pre-transplant imaging and LT may have contributed to the observed radiologic-pathologic discordance, although both methodological choices reflect routine clinical practice. Furthermore, our study lacks waitlist dropout data for non-transplanted patients, precluding an intention-to-treat analysis and limiting our conclusions to candidates who successfully reached transplantation. In conclusion, these data support the safety and feasibility of TARE and SBRT in current transplant pathways for HCC, highlighting the challenge of interpreting post-radiation treatment response within increasingly complex, multimodal treatment landscapes.

## Data Availability

All key data recorded during this study are included in this published article and its Supplementary Material. Additional raw data are available from the corresponding author on reasonable request.

## Supplementary material

SUPPLEMENTARY FIGURE S1 Scatter plots illustrating Pearson’s correlation between radiologic and pathologic TTV in the **(A)** TARE and **(B)** SBRT treatment subgroups. Solid lines represent linear regression for each cohort.
