# Supplementary material for: Stereotactic body radiation therapy and radioembolization as bridging strategies to liver transplantation: a retrospective real-world analysis
Source: Transpl Int. 2026 Oct 14;39:17047. doi: 10.3389/ti.2026.17047 (PMC13713436; doi:10.3389/ti.2026.17047)
Supplement: Supplementary file 1 [file Table1.docx]

**Table S1: Demographics**

| **Demographic variables** | **Y90 (n=23)** | **SBRT (n=33)** | **p-value** |
| --- | --- | --- | --- |
| **Age**  mean±SD | 61.78 ± 6.60 | 60.73 ± 6.98 | 0.57 (t) |
| **Sex (Male)** | 20 (86.96%) | 29 (87.88%) | 1.00 (f) |
| **Cirrhosis** | 23 (100.00%) | 30 (90.91%) | 0.26 (f) |
| **Background liver disease** |  |  | 0.97 (f) |
| - Viral | 14 (60.87%) | 22 (66.67%) |  |
| - EtOH | 3 (13.04%) | 4 (12.12%) |  |
| - MASLD/MASH | 4 (17.39%) | 4 (12.12%) |  |
| - Other | 2 (8.70%) | 3 (9.09%) |  |
| **Child-Pugh score**  Median (IQR) | 5 (5, 6) | 5 (5, 7) | 0.66 (w) |
| **MELD (pre-LRT)**  Median (IQR) | 8 (7, 11) | 8 (7, 11) | 0.85 (w) |
| **Portal hypertension** | 13 (56.52%) | 22 (68.75%) | 0.40 (f) |
| **Encephalopathy** | 8 (34.78%) | 5 (15.62%) | 0.12 (f) |
| **Ascites** | 10 (43.48%) | 12 (36.36%) | 0.78 (f) |
| **Portal vein thrombosis** | 5 (21.74%) | 3 (9.09%) | 0.25 (f) |
| **TIPS** | 0 (0.00%) | 1 (3.03%) | 1.00 (f) |
| **Diabetes** | 8 (34.78%) | 13 (39.39%) | 0.78 (f) |
| **Obesity** | 7 (30.43%) | 8 (24.24%) | 0.76 (f) |
| **Charlson Comorbidity Index**  mean±SD | 6.17 ± 1.77 | 6.55 ± 1.50 | 0.42 (t) |

**Table S2: Tumor Characteristics at LTR**

| **Variable** | **Y90 (n=23)** | **SBRT (n=33)** | **p-value** |
| --- | --- | --- | --- |
| **Number of tumors**  Median (IQR) | 2.0 (1.0, 3.0) | 2.0 (1.0, 2.0) | 0.39 (w) |
| **Diameter of biggest tumor,** cm  Median (IQR) | 3.80 (2.90, 4.80) | 3.20 (2.10, 3.60) | 0.014 (w) |
| **TTV,** cm^3^  Median (IQR) | 29.64 (15.59, 71.60) | 17.85 (4.85, 27.0) | 0.009 (w) |
| **AFP,** mcg/L  Median (IQR) | 10.00 (5.00, 53.00) | 12.0 (4.15, 63.65) | 0.69 (w) |
| **Within Milan criteria** | 10 (43.48%) | 23 (69.70%) | 0.06 (f) |
| **Within Ontario criteria^#^** | 21 (91.30%) | 30 (90.91%) | 1.00 (f) |

**Table S3: Prior LRT Therapies (before radiation)**

| **Variable** | **Y90 (n=23)** | **SBRT (n=33)** | **p-value** |
| --- | --- | --- | --- |
| **Systemic** |  |  | 0.060 (f) |
| - Sorafenib | 1 (4.35%) | 0 (0.00%) |  |
| - Lenvatinib | 2 (8.70%) | 0 (0.00%) |  |
| **Embolic** |  |  | 0.042 (f) |
| - TACE | 2 (8.70%) | 10 (30.30%) |  |
| - Bland embolization | 0 (0.00%) | 2 (6.06%) |  |
| **Ablation** | 5 (21.74%) | 18 (54.55%) | 0.026 (f) |
| **Radiation** | 0 (0.00%) | 1 (3.03%) | 1.00 (f) |
| **Resection** | 3 (13.04%) | 6 (18.18%) | 0.85 (f) |
| **Overall** | 8 (34.78%) | 26 (78.79%) | 0.0019(f) |

**Table S4: Tumor response post-LRT**

| **Variable** | **Y90 (n=23)** | **SBRT (n=33)** | **p-value** |
| --- | --- | --- | --- |
| **Number of tumors**  Median (IQR) | 1.0 (0.0, 2.0) | 1.0 (0.5, 2.0) | 0.80 (w) |
| **TTV,** cm^3^  Median (IQR) | 7.34 (0.0, 17.74) | 3.05 (0.0, 9.76) | 0.53 (w) |
| **LI-RADS Treatment Response** |  |  | 0.35 (c) |
| - Non-viable | 12 (52.17%) | 19 (57.58%) |  |
| - Nonprogressing | 4 (17.39%) | 9 (27.27%) |  |
| - Viable | 7 (30.43%) | 5 (15.15%) |  |
| **Within Milan criteria** | 18 (78.26%) | 28 (84.85%) | 0.72 (f) |
| **Within Ontario criteria^#^** | 23 (100.00%) | 33 (100.00%) | 1.00 (f) |
| **MELD (post-LRT)**  Median (IQR) | 11 (8, 16) | 10 (8, 13) | 0.65 (w) |
| **RILD** | 0 (0.00%) | 1 (3.03%) | 1.00 (f) |

**Table S5: Tumor characteristics before transplant**

| **Variable** | **Y90 (n=23)** | **SBRT (n=33)** | **p-value** |
| --- | --- | --- | --- |
| **Number of tumors**  Median (IQR) | 1.0 (0.0, 2.0) | 1.0 (0.0, 2.0) | 0.23 (w) |
| **Diameter of biggest tumor**, cm  Median (IQR) | 2.3 (1.5, 3.0) | 2.5 (1.8, 3.4) | 0.65 (w) |
| **TTV,** cm^3^  Median (IQR) | 1.77 (0.0, 8.39) | 3.05 (0.0, 21.71) | 0.39 (w) |
| **AFP,** mcg/L  Median (IQR) | 5.0 (3.9, 27.1) | 6.8 (2.4, 26.0) | 0.40 (w) |

**Table S6: Surgical Data**

| **Variable** | **Y90 (n=23)** | **SBRT (n=33)** | **p-value** |
| --- | --- | --- | --- |
| **DCD graft** | 5 (21.74%) | 6 (18.18%) | 0.75 (f) |
| **Length of stay,** days  Median (IQR) | 10 (7, 15) | 13 (8, 18) | 0.10 (w) |
| **ICU length of stay,** days  Median (IQR) | 2 (1, 2) | 2 (1, 4) | 0.47 (w) |
| **Acute rejection** | 1 (4.35%) | 4 (12.12%) | 0.64 (f) |
| **Biliary stricture** | 2 (8.70%) | 3 (9.09%) | 1.00 (f) |
| **Bile leak** | 2 (8.70%) | 2 (6.06%) | 1.00 (f) |
| **Hepatic artery thrombosis** | 1 (4.35%) | 1 (3.03%) | 1.00 (f) |
| **Reoperation** | 3 (13.04%) | 7 (21.21%) | 0.50 (f) |
| **Re-transplantation** | 0 (0.00%) | 1 (3.03%) | 1.00 (f) |

**Table S7: Pathology Data**

| **Variable** | **Y90 (n=23)** | **SBRT (n=33)** | **p-value** |
| --- | --- | --- | --- |
| **TTV (viable),** cm^3^  Median (IQR) | 41.85 (15.60, 107.90) | 12.80 (4.35, 40.60) | 0.023 (w) |
| **Complete response** | 5 (21.74%) | 12 (36.36%) | 0.376 (f) |
| **Extrahepatic disease** | 2 (9.09%) | 1 (3.23%) | 0.563 (f) |
| **Microvascular invasion** | 6 (28.57%) | 5 (15.62%) | 0.310 (f) |
| **Macrovascular invasion** | 1 (4.55%) | 1 (3.12%) | 1.000 (f) |

**Notes**

1. Continuous variables are presented as Mean ± Standard Deviation OR Median (Interquartile Range)
2. Categorical variables are presented as n (%)
3. Missing data are explicitly reported for all variables with incomplete observations
4. Percentages are calculated based on valid observations unless otherwise specified
1. (f): Fisher exact test; (c): Chi square test; (t): two sample t test; (w): Wilcoxon rank sum test
2. ^#^Ontario criteria defined by a TTV < 145 cm^3^ and an AFP < 1000 mcg/L

**Abbreviations**

AFP (*Alpha-Fetoprotein*), DCD (*Donation after Circulatory Death*), EtOH (*Ethanol*), ICU (*Intensive Care Unit*), IQR (*InterQuartile Range*), LI-RADS (*Liver Imaging-Reporting and Data System*), MASH (*Metabolic dysfunction-Associated SteatoHepatitis*), MASLD (*Metabolic dysfunction-Associated Steatotic Liver Disease*), MELD (*Model for End-Stage Liver Disease*), RILD (*Radiation-Induced Liver Disease*), SD (*Standard Deviation*), TACE (*Transarterial Chemoembolization*), TIPS (*Transjugular Intrahepatic Portosystemic Shunt*), TTV (*Total Tumor Volume*)
